# Arenavirus Diversity and Phylogeography of *Mastomys natalensis* Rodents, Nigeria

**DOI:** 10.3201/eid2204.150155

**Published:** 2016-04

**Authors:** Ayodeji Olayemi, Adeoba Obadare, Akinlabi Oyeyiola, Joseph Igbokwe, Ayobami Fasogbon, Felix Igbahenah, Daniel Ortsega, Danny Asogun, Prince Umeh, Innocent Vakkai, Chukwuyem Abejegah, Meike Pahlman, Beate Becker-Ziaja, Stephan Günther, Elisabeth Fichet-Calvet

**Affiliations:** Obafemi Awolowo University, Ile-Ife, Nigeria (A. Olayemi, A. Obadare, A. Oyeyiola, J. Igbokwe);; Ambrose Alli State University, Ekpoma, Nigeria (A. Fasogbon);; Benue State University, Makurdi, Nigeria (F. Igbahenah, D. Ortsega);; Irrua Specialist Teaching Hospital, Irrua, Nigeria (D. Asogun, C. Abejegah);; Nigerian Montane Forest Project, Ngel-Nyaki, Nigeria (P. Umeh);; Federal Medical Centre, Jalingo, Nigeria (I. Vakkai);; Bernhard Nocht Institute for Tropical Medicine, Hamburg, Germany (M. Pahlman, B. Becker-Ziaja, S. Günther, E. Fichet-Calvet)

**Keywords:** Lassa, *Mastomys natalensis*, arenaviruses, Nigeria, West Africa, phylogeography, rodents, lineages, phylogroups, hemorrhagic fever, viruses

## Abstract

*Mastomys natalensis* rodents are natural hosts for Lassa virus (LASV). Detection of LASV in 2 mitochondrial phylogroups of the rodent near the Niger and Benue Rivers in Nigeria underlines the potential for LASV emergence in fresh phylogroups of this rodent. A Mobala-like sequence was also detected in eastern Nigeria.

Lassa fever, a viral hemorrhagic disease, is estimated to infect 150,000–300,000 persons every year, killing ≈5,000 (*1*). Within West Africa, Lassa fever is endemic to 2 regions: 1) Guinea, Sierra Leone, and Liberia; and 2) Nigeria. Even within most of these countries, Lassa fever is endemic to certain areas but rare or completely absent in others (*2*). Zoonotic disease nidality describes the phenomenon in which geographic occurrence of a zoonotic disease is markedly focused or fragmented, as opposed to occurring continuously or spreading in a consistent pattern (*3*). Zoonotic disease nidality might result when only select phyletic groups in a host species are capable of serving as reservoirs for the pathogen (*4*).

The natural host for Lassa virus (LASV), the arenavirus that causes Lassa fever, is the multimammate rat *Mastomys natalensis* (*5*). This rodent, which is distributed all over sub-Saharan African, is also host to other arenaviruses such as the Mopeia virus in southeastern Africa (*6*), Morogoro and Gairo viruses in Tanzania (*7*,*8*), and Luna virus in Zambia (*9*).

In a genetic study of *M. natalensis* rodents across Africa (*10*), which analyzed cytochrome *b* sequences, researchers found that populations of these rodents in western Africa belong to the same monophylogenetic phylogroup, A-I. However, those authors detected phylogroup A-I of *M. natalensis* rodents in countries west of Nigeria and phylogroup A-II in countries east of Nigeria, but they did not sample Nigeria, the contact zone for rodents of these phylogroups. As a country in which Lassa fever is endemic in the western and eastern areas (*2*), Nigeria presents an excellent opportunity for investigation of patterns of LASV and arenavirus occurrence in 2 phylogroups of *M. natalensis* rodents. Our objectives in this study were to 1) determine which *M. natalensis* rodent cytochrome *b* phylogroups (A-I and A-II) are infected with LASV and other arenaviruses, and 2) identify the limits of distribution of these phylogroups within Nigeria.

## The Study

**Figure 1:**
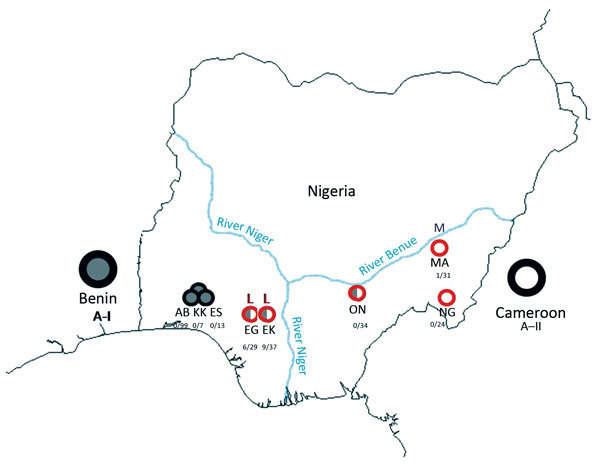
Sites at which *Mastomys natalensis* rodents were captured in Nigeria during January 2011–March 2013. Red circles represent sites within the Lassa fever–endemic zone; black circles represent sites outside the Lassa fever–endemic zone. Within the circles, gray indicates *M. natalensis* phylogroup A-I rodents; white indicates *M. natalensis* phylogroup A-II rodents; both colors within 1 circle indicate that rodents of both phylogroups were present at that site. Numbers under each site indicate number of arenavirus-positive *M. natalensis* rodents/number of *M. natalensis* captured. L indicates sites with Lassa virus–positive *M. natalensis* rodents; M indicates sites with Mobala-like virus–positive *M. natalensis* rodents. AB, Abagboro 07°32′38.0′′N, 04°30′47.2′′E; KK, Kako 07°41′26.3′′N, 04°37′09.8′′E; ES, Esira 07°42′04.7′′N, 04°39′19.4′′E; EG, Eguare-Egoro 06°46′22.7′′N, 06°05′32.5′′E; EK, Ekpoma 06°44′29.1′′N, 06°06′17.6′′E; ON, Onmba-Abena 07°38′27.5′′N, 08°24′23.6′′E; MA, Mayo-Ranewo 08°49′27.2′′N, 10°55′15.2′′E; NG, Ngel-Nyaki 07°05′30.8′′N, 11°05′27.9′′E.

From January 2011 through March 2013, small mammals were captured in H.B. Sherman live animal traps (https://www.shermantraps.com/) at 8 sites across Lassa fever–endemic and –nonendemic areas in Nigeria (Figure 1). We classified Lassa fever–nonendemic areas as areas where no cases of Lassa fever have been documented (*2*). Permission to trap rodents in various localities was granted by the Ministry of Environment, Osun State; Gwer West Local Government Council, Benue State; and the Ministry of Health, Taraba State.

Among 782 small mammals, 274 *M. natalensis* rodents were trapped. Identification of the animals in the field was based on external morphology and later confirmed genetically by cytochrome *b* gene sequencing. The rodents were euthanized, and biopsy samples (blood, liver, kidneys, spleen) were collected for laboratory analyses. Precautions for working with animals potentially infected with dangerous pathogens were strictly followed (*11*).

Using a QIAamp Viral RNA Mini Kit (QIAGEN, Valencia, CA, USA), we extracted total RNA from 20 μL of whole blood frozen at −80°C. Extracted RNA was tested with a panarenavirus protocol designed to amplify the L (polymerase) gene (340 nt) (*12*) and with another reverse transcription PCR specific for LASV, selective for the glycoprotein precursor (GPC) gene (303 nt) (*13*). We conducted further PCR amplification of the GPC fragment (using primers in Technical Appendix 1 Table) for specimens positive on initial screening. Phylogenies were inferred by use of the Bayesian Markov Chain Monte Carlo method implemented in BEAST version 1.6.2 (http://beast.bio.ed.ac.uk/).

**Figure 2:**
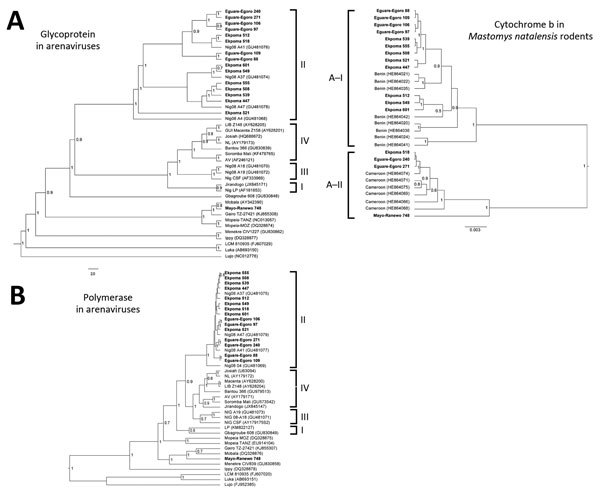
A) Phylogenetic analyses of glycoprotein precursor gene (GPC) of Old World arenaviruses and cytochrome *b* sequences of 16 arenavirus-positive *Mastomys natalensis* rodents captured in Nigeria during January 2011–March 2013 (boldface). The GPC tree (949 nt) was inferred by using the Bayesian Markov Chain Monte Carlo method, in a general time reversible plus gamma plus relaxed uncorrelated lognormal clock model. A random local clock was used for the cytochrome *b* tree. Bayesian posterior probabilities are shown at the node of the branches. The 4 lineages of the Lassa virus clade are indicated to the right of the GPC tree, and the 2 clades of *M. natalensis* rodents (A-I and A-II) are indicated on left of the cytochrome *b* tree. Scale bars indicate genetic distance. B) Phylogenetic analysis of the L (polymerase) gene in Old World arenaviruses, including the 16 new sequences found in *M. natalensis* rodents in Nigeria (boldface). The L tree (340 nt) was inferred by using the same method used for GPC analysis. GenBank numbers for reference isolate sequences are shown in parentheses.

Of the 274 *M. natalensis* rodents from the 8 sampled sites, 16 were positive by PCR for arenavirus (Figure 1; Table). Phylogenetic analyses of the GPC and L gene sequences showed that 15 of the viruses were Lassa and 1 was a Mobala-like virus (Figure 2). The LASV sequences from Ekpoma and Eguare-Egoro belonged to lineage II and clustered with strains Nig08-A4, A37, A41, and A47 from patients in Edo State (*14*). Nucleotide identities between the sequences of LASV from the rodents and those from the human patients were 82%–96% (GPC) and 85%–97% (L), and amino acid identities were 95%–99% (GPC) and 93%–100% (L), respectively (Technical Appendix 2). The sequence from Mayo Ranewo was conversely more distant from those from Mobala and Gairo; nucleotides identities were 69%–73% (GPC) and 77%–80% (L), and amino acid identities were 75%–77% (GPC) and 90%–95% (L), respectively (Technical Appendix 2).

**Table:** Source and testing results for arenavirus-positive *Mastomys natalensis* rodents, Nigeria, January 2011–March 2013*

Specimen no.	Date of capture	Village	Habitat	GPC gene (*13*)	GenBank accession no.	L gene (*12*)	GenBank accession no.
88	2011 Mar 22	Eguare-Egoro	Indoors	+	KP640562	+	KP688321
97	2011 Mar 22	Eguare-Egoro	Indoors	+	KP640563	+	KP688322
106	2011 Mar 23	Eguare-Egoro	Indoors	+	KP640564	+	KP688323
109	2011 Mar 23	Eguare-Egoro	Indoors	+	KP640565	+	KP688324
240	2011 Oct 17	Eguare-Egoro	Indoors	+	KP640566	+	KP688325
271	2011 Oct 19	Eguare-Egoro	Indoors	+	KP640567	+	KP688326
447	2012 Mar 26	Ekpoma	Peridomestic outdoor vegetation	+	KP640568	+	KP688327
508	2012 Oct 18	Ekpoma	Indoors	+	KP640569	+	KP688328
512	2012 Oct 18	Ekpoma	Peridomestic outdoor vegetation	+	KP640570	+	KP688329
518	2012 Oct 18	Ekpoma	Indoors	+	KP640571	+	KP688330
521	2012 Oct 18	Ekpoma	Peridomestic outdoor vegetation	+	KP640572	+	KP688331
539	2012 Oct 18	Ekpoma	Peridomestic outdoor vegetation	+	KP640573	+	KP688332
549	2012 Oct 18	Ekpoma	Peridomestic outdoor vegetation	+	KP640574	+	KP688333
555	2012 Oct 18	Ekpoma	Indoors	+	KP640575	+	KP688334
601	2012 Oct 21	Ekpoma	Peridomestic outdoor vegetation	+	KP640576	+	KP688335
748	2013 Mar 04	Mayo-Ranewo	Indoors	–	KP640577†	+	KP688336

*The cytochrome *b* sequences from these 16 arenavirus-positive *M. natalensis* rodents were assigned GenBank accession nos. KP688337–KP688352. +, positive; –, negative. †Sequence KP640577 was eventually obtained by using primers OWS-1 and OWS-1000 (Technical Appendix 1 Table.

Sequence analysis of the region coding cytochrome *b* indicated that *M. natalensis* rodents from Nigeria cluster in 2 clades. The first clade corresponds to phylogroup A-I, which clusters with sequences from rodents from Benin, which is west of Nigeria (Figures 1, 2; Technical Appendix 2). Phylogroup A-I, including sequences from Abagboro, Esira, Kako, Eguare-Egoro, Ekpoma from western Nigeria, extends across the Niger and Benue Rivers into Onmba-Abena in eastern Nigeria.

The second clade corresponds to phylogroup A-II, which clustered with sequences from Cameroon, which is east of Nigeria (Figures 1, 2; Technical Appendix 1 Figure). Phylogroup A-II within Nigeria is represented by *M. natalensis* rodents from Ngel-Nyaki, Mayo-Ranewo, and Onmba-Abena in eastern Nigeria, but this phylogroup also overlaps the Niger and Benue Rivers westward into Eguare-Egoro and Ekpoma. The contact zone between rodents of phylogroups A-I and A-II in Nigeria was detected at sites relatively close to the Niger and Benue Rivers (Eguare-Egoro, Ekpoma, Onmba-Abena) (Figure 1). The Niger River has been demonstrated to be a natural barrier for some rodents (*15*) but seems to delimit these 2 phylogroups only to an extent. Human-assisted long-distance migration of commensal rodents could influence their genetic structure, which may be what happened for rodents of the same *M. natalensis* phylogroup that were detected on opposite banks of the Niger River.

## Conclusions

*M. natalensis* phylogroup A-I rodents were infected with LASV in Eguare-Egoro and Ekpoma but not in Abagboro, Kako, and Esira. Because all rodents from these sites belong to the same phylogroup, some factor other than cytochrome *b* genetic structure might be responsible for the focal prevalence of LASV. It could be, however, that our study was limited by use of the cytochrome *b* mitochondrial marker only, which is maternally inherited. Therefore, other biparentally inherited genetic markers, such as microsatellites, should be investigated. Environmental variables such as humidity and temperature could also be considered (*2*).

*M. natalensis* phylogroup A-II rodents were infected by LASV and a Mobala-like virus. We did not detect any LASV-positive, phylogroup A-II rodents east of the Niger River, although all the sites sampled in this area lie within the Lassa fever–endemic zone and regularly experience epidemics (*2*). It is worth exploring the possibility that other small mammals might also host LASV. LASV-positive members of phylogroup A-II, however, were found on the west bank of the Niger River in Eguare-Egoro and Ekpoma (along with LASV-positive members of phylogroup A-I). A crucial implication of these findings is the potential that new, previously naive populations and phylogroups of *M. natalensis* rodents could become infected with LASV and the disease could emerge in new regions in western Africa.

Detection of the Mobala-like virus in *M. natalensis* rodents within Mayo-Ranewo in eastern Nigeria deserves further study. We included Mayo-Ranewo among our survey sites because an epidemic of hemorrhagic fever, considered but not confirmed to be Lassa fever, occurred there in 2012. Whether the Mobala-like arenavirus detected in this village has pathogenic properties remains to be determined.

**Technical Appendix 1**. Primers used for viral and cytochrome b testing and phylogenetic analysis of cytochrome *b* in study of arenavirus diversity among phylogroups of *Mastomys natalensis* rodents captured in Nigeria during January 2011–March 2013.

**Technical Appendix 2.** Identity scores for the nucleotides and amino acids of the partial sequence of glycoprotein (949 nt).
