# Supplementary material for: Arenavirus Diversity and Phylogeography of Mastomys natalensis Rodents, Nigeria
Source: Emerg Infect Dis. 2016 Apr;22(4):694–7. doi: 10.3201/eid2204.150155 (PMC4806934; doi:10.3201/eid2204.150155)
Supplement: Supplementary file 1 — Technical Appendix 1. Primers used for viral and cytochrome b testing and phylogenetic analysis of cytochrome b in study of arenavirus diversity among phylogroups of Mastomys natalensis rodents captured in Nigeria during January 2011–March 2013. [file 15-0155-Techapp-s1.pdf]

Article DOI: http://dx.doi.org/10.3201/eid2204.150155

Arenavirus Diversity among Phylogroups of *Mastomys natalensis* Rodents, Nigeria

Technical Appendix 1

Technical Appendix 1 Table. Primers used for viral and cytochrome b testing

Reference	Forward, 5′→3′	Reverse, 5′→3′
(*1*)	LVL3359A: AGAATTAGTGAAAGGGAGAGCAATTC<br>LVL3359D: AGAATCAGTGAAAGGGAAAGCAATTC<br>LVL3359G: AGAATTAGTGAAAGGGAGAGTAACTC	LVL3754A: CACATCATTGGTCCCCATTTACTATGATC<br>LVL3754D: CACATCATTGGTCCCCATTTACTGTGATC
(*2*)	LVS-39: ACC GGG GAT CCT AGG CAT TT	LVS-339: GTT CTT TGT GCA GGA (AC)AG GGG CAT (GT)GT CAT
(3)	OWS-1: GCGCACCGGGGATCCTAGGC	OWS-1000: AGCATGTCACAAAAYTCYTCATCATG
(*4*)	L7: ACC AAT GAC ATG AAA AAT CAT CGT T	H15915: TCT CCA TTT CTG GTT TAC AAG AC

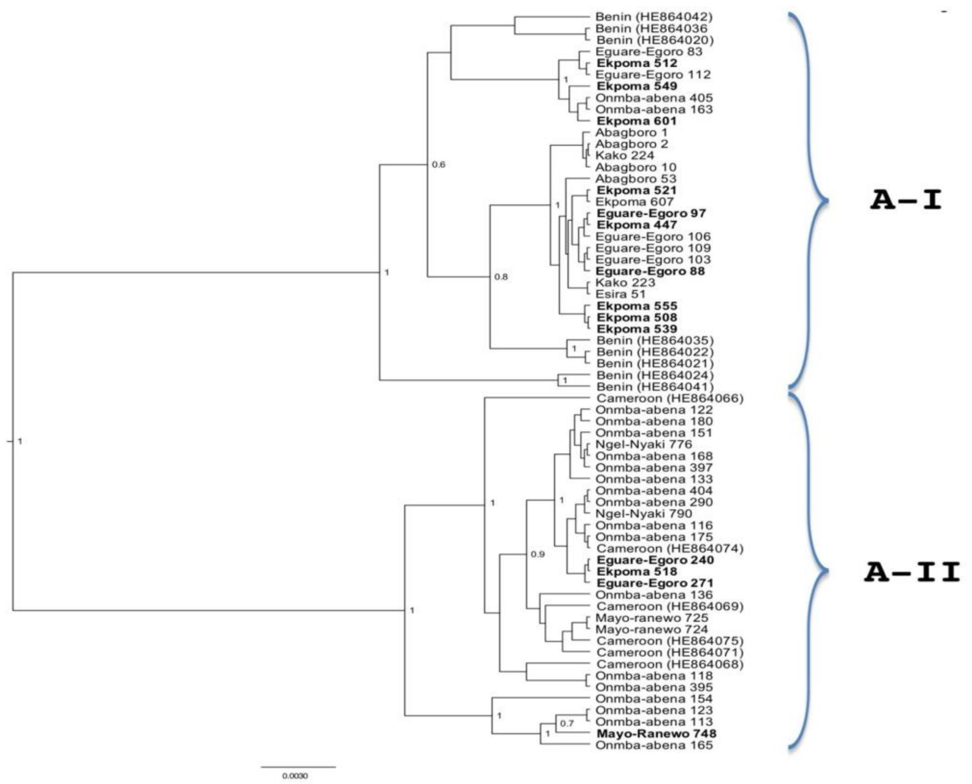

Technical Appendix 1 Figure. Phylogenetic analysis of cytochrome *b* (900 nt) of arenaviruses from *Mastomys natalensis* rodents caught in the 8 localities in Nigeria, compared to those already described in Benin and in Cameroun. Hybrid zone between clades A-I and A-II appears to be located along Niger and Benue Rivers, since both genogroups exist in Ekpoma/Eguare-Egoro and Onmba-Abena. Boldface indicaters arenavirus-positive specimens. Scale bars represent genetic distance.

References

1. Vieth S, Drosten C, Lenz O, Vincent M, Omilabu S, Hass M, et al. RT-PCR assay for detection of Lassa virus and related Old World arenaviruses targeting the L gene. Trans R Soc Trop Med Hyg. 2007;101:1253–64. http://dx.doi.org/10.1016/j.trstmh.2005.03.018 PubMed

2. Olschläger S, Lelke M, Emmerich P, Panning M, Drosten C, Hass M, et al. Improved detection of Lassa virus by reverse transcription-PCR targeting the 5′ region of S RNA. J Clin Microbiol. 2010;48:2009–13. http://dx.doi.org/10.1128/JCM.02351-09 PubMed

3. Ehichioya DU, Hass M, Becker-Ziaja B, Ehimuan J, Asogun DA, Fichet-Calvet E, et al. Current molecular epidemiology of Lassa virus in Nigeria. J Clin Microbiol. 2011;49:1157–61. http://dx.doi.org/10.1128/JCM.01891-10 PubMed

4. Ducroz J-F, Volobouev V, Granjon L. An assessment of the systematics of arvicanthine rodents using mitochondrial DNA sequences: evolutionary and biogeographical implications. J Mamm Evol. 2001;8:173–206. http://dx.doi.org/10.1023/A:1012277012303
